# The mechanisms of gastric mucosal injury: focus on initial chief cell loss as a key target

**DOI:** 10.1038/s41420-023-01318-z

**Published:** 2023-01-24

**Authors:** Zilin Deng, Jiaxing Zhu, Zhiyuan Ma, Zhiqiang Yi, Biguang Tuo, Taolang Li, Xuemei Liu

**Affiliations:** 1 Department of Gastroenterology, Digestive Disease Hospital, Affiliated Hospital of Zunyi Medical University, Zunyi, Guizhou Province China; 2 Department of General Surgery, Affiliated Hospital of Zunyi Medical University, Zunyi, Guizhou Province China

**Keywords:** Mechanisms of disease, Cancer models

## Abstract

Diffuse gastric mucosal injury is a chronic injury with altered cell differentiation, including spasmolytic polypeptide expression metaplasia (SPEM) and intestinal metaplasia (IM), which are considered precancerous lesions of gastric cancer (GC). Previously, most studies have focused on how parietal cell loss causes SPEM through transdifferentiation of chief cells. In theory, alteration or loss of chief cells seems to be a secondary phenomenon due to initial partial cell loss. However, whether initial chief cell loss causes SPEM needs to be further investigated. Currently, increasing evidence shows that initial chief cell loss is sufficient to induce gastric mucosal injury, including SPEM and IM, and ultimately lead to GC. Therefore, we summarized the two main types of models that explain the development of gastric mucosal injury due to initial chief cell loss. We hope to provide a novel perspective for the prevention and treatment of diffuse gastric mucosal injury.

## Introduction

Gastric mucosal injury can be divided into two types: focal and diffuse. Generally, the common causes of focal injury are toxin ingestion, bile reflux, and certain infectious agents, which result in focal erosions or full-thickness ulcerations that are rapidly repaired by increased proliferation in neighboring units and migration of surface cells; these processes eventually reestablish normal differentiation in damaged units. Diffuse gastric mucosal injury is a chronic injury with altered cell differentiation, including spasmolytic polypeptide expression metaplasia (SPEM) and intestinal metaplasia (IM), which are considered precancerous lesions of gastric cancer (GC) [1–6].

Previously, most studies focused on parietal cell loss as the initial factor inducing diffuse gastric mucosal injury, with alteration or loss of chief cells following. These mouse models of metaplasia have been generated by gene knockout (KO) and treatment with various drugs (DMP-777, L635, and high-dose tamoxifen (HDT)), as well as *Helicobacter pylori* (*H. pylori*) infection [7–11]. However, some reports have shown that chief cell ablation that is not secondary to parietal cell loss can also cause diffuse gastric mucosal injury [12–17], suggesting that chief cell loss might be a promising strategy for treating gastric mucosal injury. This review summarizes gene KO and drug treatment strategies to induce initial chief cell deficiency and thus gastric mucosal injury and the regulatory mechanisms in gastric mucosal diseases. We hope to provide new perspectives for preventing and treating such diseases.

## The expression pattern and function of chief cells in the stomach

Chief cells reside at the base of oxyntic glands in the gastric corpus. These cells not only secrete pepsinogen and participate in digestive functions in normal gastric physiology [18] but also act as reserve stem cells that can function in response to cell loss during homeostasis or after injury [16, 19, 20].

In most previous studies, after injury to the gastric epithelium, especially after the loss of parietal cells, chief cells undergo metaplasia that causes them to transition into mucous cells, a process called SPEM. Thus, chief cells have been considered the origin of metaplasia after gastric mucosal injury. These cells usually undergo metaplasia by transdifferentiation or dedifferentiation [21, 22]. However, increasing data suggest that initial loss of chief cells (independent of parietal cell loss) causes SPEM and malignant pathological processes, providing promising targets for understanding the mechanisms of gastric mucosal injury.

In the chief cells of the stomach, multiple markers with various functions have been identified. Basic helix-loop-helix family member a15 (BHLHA15, also called Mist1) is a marker of chief cell health and maturation and is mainly expressed within chief cells. Its expression is lost in metaplasia, atypical hyperplasia and various carcinomas, providing evidence that metaplasia originates from mature chief cells [23]. Leucine-rich repeat-containing G protein-coupled receptor 5 (Lgr5) is a gastric stemness marker and is responsible for tissue renewal in the gastric epithelium [24]. Notably, a subset of Lgr5-labeled chief cells function as reserve stem cells (RSCs) in the gastric body, contributing to the renewal and regeneration of gastric epithelial tissue [16], as do Troy^+^ chief cells [20]. Acidic mammalian chitinase (CHIA) is a member of the 18-glycosidase family. It is localized in gastric chief cells [25] and is significantly downregulated in chronic atrophic gastritis (CAG) [26]. Furthermore, our laboratory demonstrated that CHIA is essential for maintaining gastric chief cell survival and is involved in diffuse gastric mucosal injury (unpublished data).

Chief cells responding to mucosal injury are subject to many regulatory mechanisms, such as maturation and aging [27, 28], switching between homeostatic chief cells and injury-responsive chief cells depending on the cell cycle regulatory protein p57 [29], initiation of the autophagic pathway and lysosomal pathway by solute carrier family 7 member 11 responsible for reverse cystine/glutamate transport (SLC7A11; also known as NAS xCT) to promote reprogramming [30] and chief cell transdifferentiation through aberrant DNA methylation caused by miRNAs [31]. Thus, it is clear that the role of chief cells in the gastric epithelium is important and complex.

## Establishment of chief cell loss-induced mucosal injury models and the relationship of chief cell loss-induced mucosal injury with the development of gastric mucosal diseases

The loss of chief cells results in various types of gastric diffuse mucosal injury, epithelial structural disorders and types of gastric metaplasia, classic precancerous lesions [12–17], including SPEM (with predominant expression of Tff2 and Muc6) and IM (with the expression of CDX2, Muc2, and Tff3) [32], which ultimately develop into GC. A recent report showed that chief cells are prone to carcinogenesis and can serve as the origin of GC [16]. Furthermore, one study confirmed by Monocle pseudotime trajectory analysis that metaplastic cells developed due to transdifferentiation of chief cells and likewise suggested that chief cells might be the origin of gastric tumor cells [33].

These data suggest that the initial loss of chief cells is sufficient to cause gastric mucosal injury, providing promising research directions for understanding the mechanisms of mucosal injury. Currently, two types of mouse models with initial chief cell loss have been established. The first involves KO of genes that are highly expressed in chief cells [12–14]. The second involves a combination of drug treatments and gene editing in mice [15–17]; this method leads to the ablation of chief cells expressing a specific gene, facilitating the study of the particular role of a small group of chief cells. The details are summarized in Table 1.

**Table 1:** Pathological lesions in mice model of chief cell loss.

Mice model of chief cell deletion	Parietal cell deletion	SPEM	IM	Gastric cancer
Gene knockout (KO)				
Runx3 KO mice model [12]	NO	YES	YES	YES
Chia KO mice model [13, 14]	NO	YES	YES	YES
Gene editing with drug treatment				
Lgr5-DTR-GFP mice treat with Diphtheria toxin [15]	NO	YES	NO	NO
Mist1-CreERT; R26-DTA mice treat with Tamoxifen [15]	NO	YES	NO	NO
Lgr5-DTR-EGFP mice treat with Diphtheria toxin [16]	NO	YES	NO	NO
Lgr5-2A-CreER-T2^tg/tg^/LSL-Kras(G12D)^tg/+^ mice treat with Tamoxifen [16]	NO	YES	NO	YES
Gpr30-rtTA;TetO-KrasG12D-IRES-TdTomato mice treat with Doxycycline [17]	NO	YES	NO	NO

### Gene deletion-induced chief cell loss results in gastric mucosal injury

RUNX3 is considered a tumor suppressor in GC [34] and is strongly expressed in chief cells, as well as in surface mucous cells [35]. Ito K et al. established a Runx3 KO mouse model in which significant loss of gastric epithelial chief cells, but not total chief cells, was observed. This loss was accompanied by the appearance of SPEM cells expressing Tff2 and IM cells expressing CDX2 and Muc2. Knockdown of Runx3 caused an acute precancerous lesion, a process involving Wnt signaling, as transactivation of β-catenin/TCF upregulated CDX2 [12]. Ogasawara N et al. were the first to report a significant correlation between Runx3 and pepsinogen I, suggesting that RUNX3 plays a role in GC by maintaining chief cell differentiation [36]. The Runx3 KO mouse model also developed adenocarcinoma under the induction of immunosuppressive agents [12].

CHIA is also predominantly expressed in chief cells of the stomach [25]. According to The Cancer Genome Atlas (TCGA) data, compared with healthy controls, gastric adenocarcinoma tissues had significantly downregulated expression of CHIA, suggesting a potential oncogene role in GC. However, whether initial chief cell loss causes SPEM and malignant pathological processes needs to be further investigated. Histopathological and immunohistochemistry (IHC) analyses, as well as in situ hybridization (ISH) with specific markers, were performed in CHIA wild-type mice and KO mice at different ages in our laboratory. With advanced age, CHIA-deficient mice exhibited a severe gastric preneoplasia phenotype, including chief cell loss followed by chronic inflammation, SPEM, and high-grade intraepithelial neoplasia (HGIN). However, mild parietal cell loss was found only in the HGIN stage. Furthermore, deletion of CHIA in chief cells resulted in upregulation of specific markers of SPEM, including Tff2 and Muc6. ISH and IHC analyses showed that loss of CHIA not only significantly altered multiple markers of gastric epithelial cell differentiation but also progressively and significantly reduced gastric stem cell (GSC) markers, including Lrg5 and Mist1, from 8 days to 15 months after birth. However, CHIA deletion in mice resulted in a mild decrease in Lrig1, a marker of parietal cell differentiation of GSCs, as well as parietal cell markers, including H^+^/K^+^-ATPase, Slc26a9 and Sonic hedgehog. These changes were observed only at the HGIN stage, not in the SPEM phase (Zhao et al. unpublished data) [13, 14].

In conclusion, the loss of chief cells should be noted in diffuse gastric mucosal injury. An independent origin of malignant events in the gastric epithelium unrelated to the absence of parietal cells and infection with *H. pylori* should be considered.

### Drug-induced chief cell loss results in gastric mucosal injury

Drug-induced initial chief cell loss also causes gastric mucosal injury. Researchers administered diphtheria toxin and small doses of tamoxifen to ablate Lgr5^+^ chief cells and Mist1^+^ chief cells, and both agents induced short-term SPEM without affecting parietal cells, accompanied by rapid, short-term expansion of GSII^+^ GIF^+^ cells [15]. Moreover, Leushacke M et al. similarly used diphtheria toxin to ablate chief cells labeled by Lgr5. The glandular structure showed significant disruption and extensive apoptosis, including a reduction in basal chief cells and surface mucous cells, suggesting that long-term ablation of Lgr5-expressing chief cells impairs epithelial homeostasis [16].

Activation of Kras has been suggested to be a key signal in the development of GC [37–39], and systemically induced activation of Kras has also been shown to lead to metaplasia in the stomach [40]. Leushacke M’s group used tamoxifen to induce Kras activation in Lgr5^+^ chief cells, and mouse glands also showed metaplasia. These metaplastic glands showed strong expression of Tff2 and Muc5ac and the absence of parietal cells, as well as macrophage infiltration, consistent with SPEM features [16]. Moreover, Hata M’s group established a mouse model targeting Gpr30^+^ chief cells with Kras mutations, since they found that Gpr30 is widely and specifically expressed in chief cells and can be used as a chief cell-specific marker. After prolonged Kras induction by doxycycline, the chief cells were lost as expected, while the parietal cells remained viable. These effects were accompanied by the appearance of a GIF^+^GSII^+^ SPEM lineage, which resulted from regenerative expansion of Kitl^+^ isthmus progenitors in response to the loss of chief cells rather than transdifferentiation or dedifferentiation of Gpr30^+^ chief cells. Chief cells are subject to Gpr30 regulation by rapid ablation from epithelial cells through PDK-dependent cell competition. These effects may occur via induction of the Warburg effect caused by enhanced PDH phosphorylation in Gpr30^+^ chief cells [17]. It is also possible that the direct binding of estrogen receptors to tamoxifen triggers PDK-dependent metabolic changes and promotes cellular competition [41].

These findings suggest that both gene deletion- and drug-induced initial chief cell loss are also origins of gastric mucosal injury and not just effects of parietal cell loss.

## Opinions and outlook

The gastric mucosal barrier provides protection for the stomach, and an imbalance between invasive and protective factors can lead to the development of gastric mucosal disease. Most of the previous studies on gastric mucosal diseases have focused on parietal cells. However, in-depth research on mucosal diseases has revealed that chief cells should also be emphasized in the prevention and treatment of gastric mucosal injury. The initial loss of chief cells is an independent key event in gastric mucosal injury and in the development of gastric precancerous lesions and gastric tumors. This review provides a new perspective on gastric mucosal diseases, furthering the understanding of the role of chief cells in these diseases and providing new targets for prevention and treatment.

## Data Availability

All the data used to support the findings of this study are available in the paper.
